# Development of a qualitative real-time PCR method to detect 19 targets for identification of genetically modified organisms

**DOI:** 10.1186/s40064-016-2395-y

**Published:** 2016-06-24

**Authors:** Cheng Peng, Pengfei Wang, Xiaoli Xu, Xiaofu Wang, Wei Wei, Xiaoyun Chen, Junfeng Xu

**Affiliations:** Institute of Quality and Standard for Agro-Products, Zhejiang Academy of Agricultural Sciences, Hangzhou, 310021 China; State Key Laboratory Breeding Base for Zhejiang Sustainable Pest and Disease Control, Hangzhou, 310021 China; College of Agriculture and Biotechnology, Zhejiang University, Hangzhou, 310058 China

**Keywords:** Genetically modified organism (GMO), Identification, Screening, Real-time PCR

## Abstract

As the amount of commercially available genetically modified organisms (GMOs) grows recent years, the diversity of target sequences for molecular detection techniques are eagerly needed. Considered as the gold standard for GMO analysis, the real-time PCR technology was optimized to produce a high-throughput GMO screening method. With this method we can detect 19 transgenic targets. The specificity of the assays was demonstrated to be 100 % by the specific amplification of DNA derived from reference material from 20 genetically modified crops and 4 non modified crops. Furthermore, most assays showed a very sensitive detection, reaching the limit of ten copies. The 19 assays are the most frequently used genetic elements present in GM crops and theoretically enable the screening of the known GMO described in Chinese markets. Easy to use, fast and cost efficient, this method approach fits the purpose of GMO testing laboratories.

## Background

With limited crop resources and climate disorders, the total cultivated land area dedicated to genetically modified organism (GMO) has been rising for several years. By the end of 2012, a total of 170 million hectares of genetically modified (GM) crops were planted in 28 countries, 319 GM events of 25 species were approved, and 2497 regulatory approvals were issued in 59 countries (James 2012). With global expansion in the areas sown to transgenic crops, the likelihood of contamination of non-transgenic varieties with GM products is increasing. Examples include the accidental presence of herbicide tolerant rice in Europe, and insect-resistant rice containing TT51-1, KMD1, or KF6 transformation events has been found and reported in the marketplace (Akiyama et al. 2007; GeneWatch UK and Greenpeace International 2008; Rapid Alert System for Food and Feed 2010). To monitor the presence of GM ingredients, methods of GM detection that allow for proper labeling are now required in over 30 countries.

To date, a wide variety of methodologies have been developed for the monitoring of GM contents, such as two-dimensional electrophoresis (Kim et al. 2006), protein capillary electrophoresis (Latoszek et al. 2011), HPLC (López et al. 2009), and ELISA (Xu et al. 2009). Of these methodologies though, polymerase chain reaction (PCR) and real-time PCR is currently the most widely applied technique owing to its high sensitivity and specificity (Marmiroli et al. 2008) while conventional PCR methods need to handle post-PCR products for gel electrophoresis or enzymatic digestion, real-time PCR does not need post-PCR manipulations which significantly reduce the risk of laboratory contamination and is more convenient.

As the GMOs rise, the number of target sequences for molecular identification increase accordingly. Consequently, description of detection and identification PCR methods has been rising during these last few years. Many common genetic elements of GMOs such as promoters (p-35S, p-FMV), terminators (t-NOS, t-E9) or transgenes (pat, CP4epsps) has been described (Dörries et al. 2010; Guo et al. 2012). Recently, a multiplex qPCR method which was selected using minor groove binder (MGB) TaqMan^®^ probes to simultaneously detect 47 targets for the identification of genetically modified organisms has been development (Cottenet et al. 2013).

In China, to adapt the analytical approach with the growing GMO environment and to cover a wider range of GM targets, the development of a new GMO multiscreening method was undertaken. In this paper, we describe a real-time PCR screening method for GMO identification, which contains an assay for the detection of 19 important genetic elements. These sequences are often used for screening purposes in China.

## Methods

### Reference materials

GM rape: MS1, MS8, T45, Topas19/2, oxy235, RF3, RF2 and GT73. GM rice: KMD, TT51 and KF6. GM maize: NK603, BT176, TC1507, MON89034 and MON863. GM soybean: 5547-127, A2704-12, MON89788 and GTS40-3-2. All GM materials were collected by our laboratory (Hangzhou, China), and non-transgenic seeds of rape, rice, maize and soybean were also used in this study.

### DNA extraction and preparation

For all of the tested samples investigated in this study, DNA was prepared from seed flour material using a kit (DNA Extraction Kit for GMO Detection, version 3.0, Takara, Shiga, Japan). The DNA was quantified using the PicoGreen reagent according to the manufacturer’s instructions (Qubit dsDNA BR Assay Kit, Invitrogen, Shanghai, China). The purity of the extracted DNA was determined by the ratio of the absorbance at 260 and 280 nm using a spectro-photometer (Ultrospec 1100 pro, GE Healthcare, USA), and integrity was further analyzed by 1 % agarose gel electrophoresis.

### Realtime PCR

For each qPCR, 20 µl of an amplification mix consisting of 2 µl of sample DNA at 100 ng/µl, SYBR Premix Ex TaqTM (2×) 10 µl, primers (100 µmol/µl) 0.4 µl, ROX Reference DyeII (50×) 2 µl, ddH2O 5.6 µl. The real-time PCR assays were performed on an ABI7500 Real-Time Detection System (Applied Biosystems) under the following conditions: an initial denaturation step (95 °C/10 min), 45 cycles at 95 °C/10 s, and 60 °C/60 s. To comply with GMO analytical quality requirements, a negative and a positive control were analysed in parallel. In addition, *18SRNA* was used as an IPC to evaluate the absence of PCR inhibition, especially in the case of a negative result. Data were collected and processed using ABI Sequence Detection Software (version 1.4, Applied Biosystems). A positive amplification was considered when a Cq value below 38 was obtained, and the primers used in this study has been mentioned (Table 1).

**Table 1:** List of forward (F), reverse (R) primers used in this study

Assays	Primer sequences	Targeted sequences	Fragment size (bp)	References
*SPS*	F-5′-ATCTGTTTACTCGTCAAGTGTCATCTC-3′ R-5′-GCCATGGATTACATATGGCAAGA-3′	U33175, Oryza sativa sucrose phosphate synthase gene	287	Bulletin No. 1861-1-2012 of the Ministry of Agriculture of the People’s Republic of China (2012)
*Zein*	F-5′-TGAACCCATGCATGCAGT-3′ R-5′-GGCAAGACCATTGGTGA-3′	X07535, Maize delta zein structural10 gene	173	Cao et al. (2012)
*Lectin*	F-5′-GCCCTCTACTCCACCCCCATCC-3′ R-5′-GCCCATCTGCAAGCCTTTTTGTG-3′	K00821, Soybean lectin (Le1) gene	118	Bulletin No. 1485-6-2010 of the Ministry of Agriculture of the People’s Republic of China (2010)
*HMG I/Y*	F-5′-TCCTTCCGTTTCCTCGCC-3′ R-5′-TTCCACGCCCTCTCCGCT-3′	AF127919, Brassica napus high mobility group protein I/Y (HMGa) gene	206	Bulletin No. 869-4-2007 of the Ministry of Agriculture of the People’s Republic of China (2007)
*pCaMV35s*	F-5′-CCATCATTGCGATAAAGGAAA-3′ R-5′-TCATCCCTTACGTCAGTGGAG-3′	AJ783419, *pCaMV35s* element	165	Cao et al. (2012)
*FMV35S*	F-5′-AAGACATCCACCGAAGACTTA-3′ R-5′-AGGACAGCTCTTTTCCACGTT-3′	HB427176, *FMV35S* element	210	Pan et al. (2003)
*T*-*35S*	F-5′-GTTTCGCTCATGTGTTGAGC-3′ R-5′-GGGGATCTGGATTTTAGTACTG-3′	KF206153.1, *T*-*35S* element	121	Bulletin No. 1782-3-2012 of the Ministry of Agriculture of the People’s Republic of China (2012)
*T*-*E9*	F-5‘-GCCACGATTTGACACATTTTTACTC-3′ R-5′-CTGTGAAATGGAAATGGATGGAG-3′	KT388099.1, *T*-*E9* element	166	Tachezy et al. (2002)
*g7*	F-5′-AAGGCAATTTGTAGATGTTAATTCCC-3′ R-5′-ACATAATATCGCACTCAGTCTTTCATC-3′	NC_017854.1, *g7* element	197	This study
*tNOS*	F-5′-ATCGTTCAAACATTTGGCA-3′ R-5′-ATTGCGGGACTCTAATCATA-3′	AY255709, *tNOS* element	165	Cao et al. (2012)
*Cry1Ab*	F-5′-GAAGGTTTGAGCAATCTCTAC-3′ R-5′-CGATCAGCCTAGTAAGGTCGT-3′	AY326434, *Cry1Ab* gene	301	Hernandez et al. (2003), Holck et al. (2002)
*Cry2Ab*	F-5′-CAGCGGCGCCAACCTCTACG-3′ R-5′-TGAACGGCGATGCACCAATGTC-3′	NC_017203.1, *Cry2Ab* gene	260	Randhawa et al. (2010)
*NptII*	F-5′-ACCTGTCCGGTGCCCTGAATGAACTGC-3′ R-5′-GCCATGATGGATACTTTCTCGGCAGGAGC-3′	KF963132.1, *NptII* gene	196	Liu et al. (2010)
*PAT*	F-5′-CGCGGTTTGTGATATCGTTAAC-3′ R-5′-TCTTGCAACCTCTCTAGATCATCAA-3′	JX434028.1, *PAT* gene	108	Pan et al. (2003)
*bar*	F-5′-ACAAGCACGGTCAACTTCC-3′ R-5′-ACTCGGCCGTCCAGTCGTA-3′	KJ668650.1, *bar* gene	175	Pan et al. (2003)
*18S*	F-5′-CCTGAGAAACGGCTACCAT-3′ R-5′-CGTGTCAGGATTGGGTAAT-3′	KR048749.1, *18S* ribosomal RNA gene	137	Pan et al. (2012)
*HPT*	F-5′-GAAGTGCTTGACATTGGGGAGT-3′ R-5′-AGATGTTGGCGACCTCGTATT-3′	AF234296, *HPT* gene	472	Bulletin No. 1782-2-2012 of the Ministry of Agriculture of the People’s Republic of China (2012)
*barnase*	F-5′-CTGGGTGGCATCAAAAGGGAACC-3′ R-5′-TCCGGTCTGAATTTCTGAAGCCTG-3′	M14442.1, *barnase* gene	202	Delano et al. (2003)
*barstar*	F-5′-TCAGAAGTATCAGCGACCTCCACC-3′ R-5′-AAGTATGATGGTGATGTCGCAGCC-3′	AY283058.1, *barstar* gene	236	Randhawa et al. (2010)

## Results and discussion

### Specificity

To determine the specificity of the method, plant materials and GM materials with a high GM content (≥1 %) were tested in duplicate. To assess the reliability of the real-time PCR runs, a negative no template control (NTC) and a positive control were analysed in each run. All the 19 assays successfully amplified on the positive control, while no amplification curves were observed with NTC. The generic plant assay successfully amplified on all the plant species tested (Table 2) and did not lead to any signals on animal DNA (beef, pig, fish and chicken). The assays targeting soybean, maize, rice, and rapeseed were specific to their respective plant species only, genetically modified or not. No cross-reactivity was observed on any other *cry* genes such as *cry1Ab* and *cry2Ab* contained in BT176 and MON89034 GM maize events.

**Table 2:** Screening patterns obtained on reference materials for specificity testing

Tested sample	Detection element									
	*18S*	*HMG I/Y*	*SPS*	*Zein*	*Lectin*	*pCaMV35s*	*FMV35S*	*T*-*35S*	*T*-*E9*	*g7*
Non GM rape	x	x								
MS1	x	x								x
MS8	x	x								x
T45	x	x				x		x		
Topas19/2	x	x				x		x		
oxy235	x	x				x				
RF3	x	x								x
RF2	x	x								x
GT73	x	x					x		x	
Non GM rice	x		x							
KMD	x		x			x				
TT51	x		x							
KF6	x		x			x		x		
Non GM maize	x			x						
NK603	x			x		x				
BT176	x			x		x		x		
TC1507	x			x		x		x		
MON89034	x			x		x	x			
MON863	x			x		x				
Non GM soybean	x				x					
5547-127	x				x	x		x		
A2704-12	x				x	x		x		
MON89788	x				x		x		x	
GTS40-3-2	x				x	x				

Tested sample	Detection element								
	*tNOS*	*Cry1Ab*	*Cry2Ab*	*NPTII*	*PAT*	*BAR*	*HPT*	*Barnase*	*Barstar*
Non GM rape									
MS1	x			x		x		x	
MS8	x					x		x	
T45					x				
Topas19/2				x	x				
oxy235	x								
RF3	x					x			x
RF2	x			x		x			x
GT73									
Non GM rice									
KMD		x		x			x		
TT51	x	x							
KF6	x						x		
Non GM maize									
NK603	x								
BT176		x				x			
TC1507					x				
MON89034	x		x						
MON863	x			x					
Non GM soybean									
5547-127					x				
A2704-12					x				
MON89788									
GTS40-3-2	x								

An expected and an analytical positive amplification is indicated with an “X,” respectively

As previously reported the *cry1Ab* gene has been truncated and highly modified to optimise its expression in Bt176 GM maize (CERA 2013), its amplification was less efficient on Bt176 and led to higher Cq values compared to the other GM events (data not shown). Taken together, based on the theoretical transgenic construct of the tested GM events, no false-positive or false-negative signals were observed for these GM marker assays, indicating the reliable behaviour for the screening capabilities of the method.

The test results of a sample can give not only information about the general presence of a GMO but also about the identity of the GMO present or at least it gives information about necessary additional testing. Therefore a table which contains information about the screening elements of all authorized GM crops in China would be a convenient tool for routine laboratory use (Waiblinger et al. 2008). For example, the *hpt*, *NPTII* and *cry1Ab* genes were introduced in KMD GM rice as selective markers and were correctly detected. Therefore, we can separate KMD GM rice from other GM rice in our assay. In short, all the 19 assays are suitable to testing admixtures of non-transgenic and GM plants/materials, it will be useful for screening the transgenic elements in Chinese markets.

### Sensitivity

**Fig. 1:**
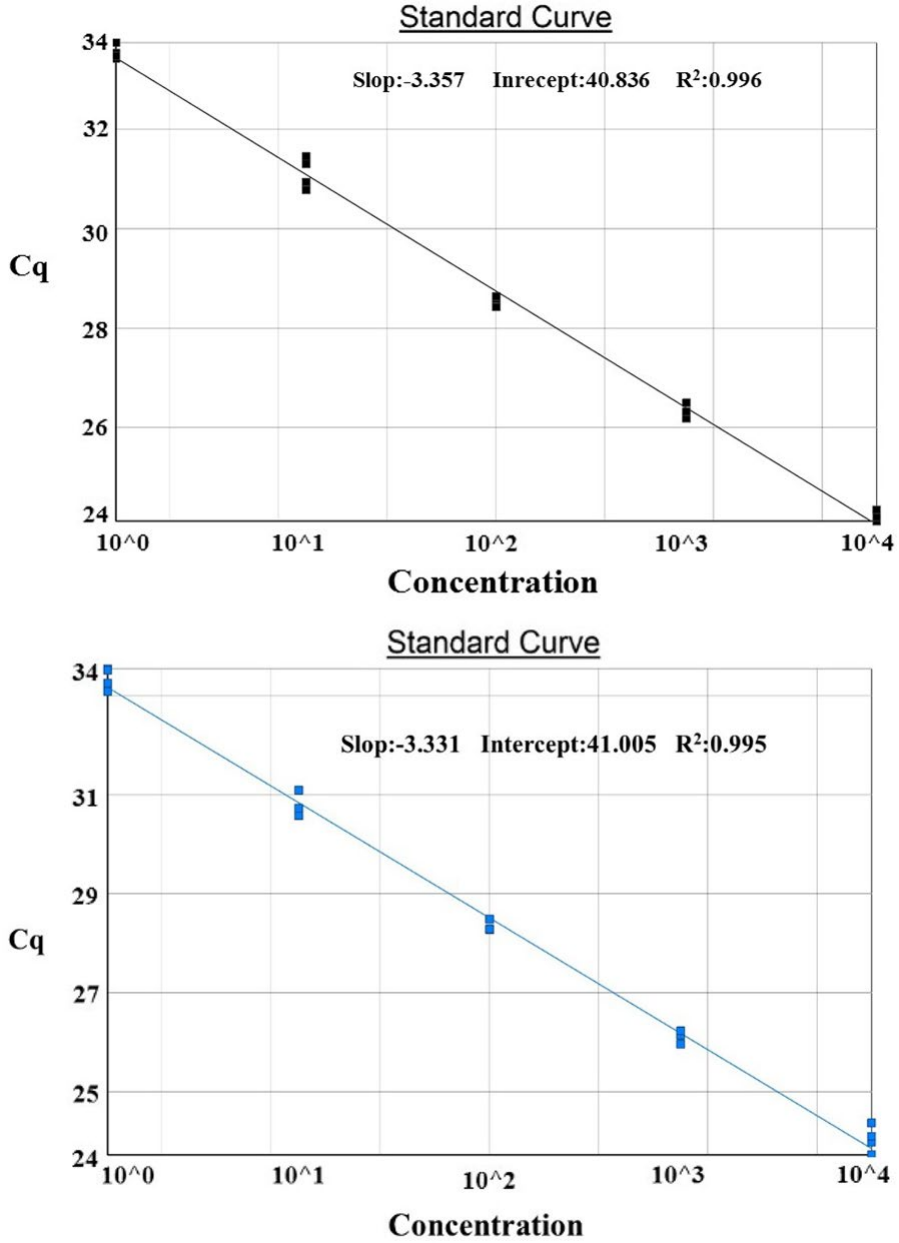
Sample dilution series on the two primers from 19 target elements selected for sensitivity analysis. The *dark* and *blue line* shows the standard curve *pCaMV35s* and *tNOS*, respectively

The LOD is defined as the lowest quantity or concentration that can be reliably detected. To determine the sensitivity of the different assays, plant materials and GM materials with a low GM content were analysed. For each target sequence, a tenfold serial dilution of known concentrations (1000–1 copy/reaction) was analyzed in triplicates, in five independent PCR runs and with three production lots of the assay components. Each point of the dilution series was therefore tested in 15 replicates. Coefficients of variation for the Cq values, ranging from 0.21 to 4.35 % for the 19 gene systems (data not shown), showed that the repeatability of the standard curves was very good. In addition, the regression analyses showed that their efficiencies were well-matched and all above 90 % (Fig. 1). With the exception of the majority of the assays reached a LOD = 0.01 % (Table 3). In addition, the absolute sensitivity (LOD_copies_) was estimated. Most assays allowed for a very sensitive detection, reaching in some cases the theoretical PCR limit of ten copies (Table 3). These results indicate that our assay was suitable for a sensitive qualitative detection of DNA derived from GMOs.

**Table 3:** LOD, LOD_copies_ and Tm value of the 19 real-time PCR assays

Detection element	Sensitivity LOD (%)	LOD_copies_	Tm value (°C)	DNA samples
*SPS*	0.01	5.53	85–86	KMD
*Zein*	0.1	4.20	85.5	Non GM maize
*Lectin*	0.1	10.39	83.6	MON89788
*HMG I/Y*	0.1	10.68	88.4	Non GM rape
*pCaMV35s*	0.01	5.53	84.2–84.5	KMD
*FMV35S*	0.1	7.56	74.8–75.5	MON89034
*T*-*35S*	0.01	5.53	75.7	Topas19/2
*T*-*E9*	0.1	10.39	76.6–77.3	MON89788
*g7*	0.01	19.45	76.6–76.9	MS1
*tNOS*	0.01	5.53	77.9–78.2	TT51
*Cry1Ab*	0.01	5.53	85.2–85.4	KMD
*Cry2Ab*	0.1	7.56	88.3–88.6	MON89034
*NptII*	0.01	5.53	87.1–87.9	KMD
*PAT*	0.1	14.27	84.5	T45
*BAR*	0.1	41.03	80.6–81	RF3
*18S*	0.01	4.20	85.1–85.8	Non GM maize
*HPT*	0.01	5.53	88.5	KMD
*Barnase*	0.1	19.45	85.6–86	MS1
*Barstar*	0.1	41.03	85.3–85.6	RF3

## Conclusion

In this study, a real-time PCR system for the simultaneous detection of 19 transgenic targets was established and showed high specificity and sensitivity. The presented qualitative real-time RCR assay offers a broad, simple and cost-efficient strategy in GMO analysis. The 19 assays are the most frequently used genetic elements present in GM crops and theoretically enable the screening of the known GMO described in Chinese markets. We believe it will be useful for screening for GMOs in the Chinese market place in the future.
